# Glutamatergic Neurons Induce Expression of Functional Glutamatergic Synapses in Primary Myotubes

**DOI:** 10.1371/journal.pone.0031451

**Published:** 2012-02-09

**Authors:** Michele Ettorre, Erika Lorenzetto, Claudia Laperchia, Cristina Baiguera, Caterina Branca, Manuela Benarese, PierFranco Spano, Marina Pizzi, Mario Buffelli

**Affiliations:** 1 Department of Neurological, Neuropsychological, Morphological and Movement Sciences, Section of Physiology and Psychology, University of Verona, Verona, Italy; 2 Department of Biomedical Sciences and Biotechnologies, University of Brescia, Brescia, Italy; 3 National Institute of Neuroscience, Rome, Italy; 4 Center for Biomedical Computing, University of Verona, Verona, Italy; 5 Istituti di Ricovero e Cura a Carattere Scientifico, S. Camillo Hospital, Venice, Italy; Emory University, United States of America

## Abstract

**Background:**

The functioning of the nervous system depends upon the specificity of its synaptic contacts. The mechanisms triggering the expression of the appropriate receptors on postsynaptic membrane and the role of the presynaptic partner in the differentiation of postsynaptic structures are little known.

**Methods and Findings:**

To address these questions we cocultured murine primary muscle cells with several glutamatergic neurons, either cortical, cerebellar or hippocampal. Immunofluorescence and electrophysiology analyses revealed that functional excitatory synaptic contacts were formed between glutamatergic neurons and muscle cells. Moreover, immunoprecipitation and immunofluorescence experiments showed that typical anchoring proteins of central excitatory synapses coimmunoprecipitate and colocalize with rapsyn, the acetylcholine receptor anchoring protein at the neuromuscular junction.

**Conclusions:**

These results support an important role of the presynaptic partner in the induction and differentiation of the postsynaptic structures.

## Introduction

The efficacy of synaptic transmission depends upon the mechanisms that regulate the assembly of the pre- and postsynaptic components. Most of our knowledge about the formation of synapses comes from studies of vertebrate neuromuscular junction (NMJ). At the NMJ, it is well established that the nerve-derived factor z-agrin, plays a predominant role in organizing numerous aspects of postsynaptic differentiation [1], [2], [3], [4]. Agrin activates LRP4/MuSK in the muscle fiber, leading to local synthesis and aggregation of acetylcholine receptors (AChRs) [5], [6], [7], [8], [9], [10], [11], [12]. Neuregulin, another molecule released from the nerve, was also proposed to induce AChRs synthesis from subsynaptic muscle nuclei through activation of ErbB receptor tyrosine kinases [13]. However, mice deficient in ErbB receptor tyrosine kinase have no defect in AChRs synthesis [14].

At central synapses, several neuronal and glial secreted synaptogenic molecules are implicated in the formation of postsynaptic structures in the central nervous system (CNS). For example EphrinB-EphB receptors, neuronal pentraxins (Narp), Cbln1, thrombospondin ([15], [16], [17], [18], [19], see also some reviews [20], [21]). Despite our knowledge about the mechanisms that regulate synaptogenesis in mammalian NMJ and interneuronal synapses, it remains largely unknown how the matching between neurotransmitter phenotype and the appropriate postsynaptic receptor is obtained in developing synapses.

Recently, it has been shown that embryonic muscle cells of Xenopus express several classes of transmitter receptors in addition to those for acetylcholine (ACh) [22]. When the presynaptic neurotransmitter is changed, by altering electrical activity, neurons can select the appropriate transmitter receptor from the population available on the surface of muscle cells [23], [24]. In another study, Brunelli et al. have shown that in a particular reinnervation model, in which descending glutamatergic fibers were diverted from the spinal cord to rat skeletal muscle by means of a peripheral nerve graft, the cholinergic synapses switch to the glutamatergic type [25], [26], [27].

In this work, to further investigate the role of presynaptic structure in the differentiation of the postsynaptic elements and to achieve the precise synaptic development, we cocultured, in separate compartments of the same plate, murine primary muscle cells with glutamatergic neurons. We found that primary glutamatergic neurons form functional glutamatergic synapses with skeletal muscle cells.

## Results

### Formation of synaptic contacts between glutamatergic neurons and muscle cells

**Figure 1:**
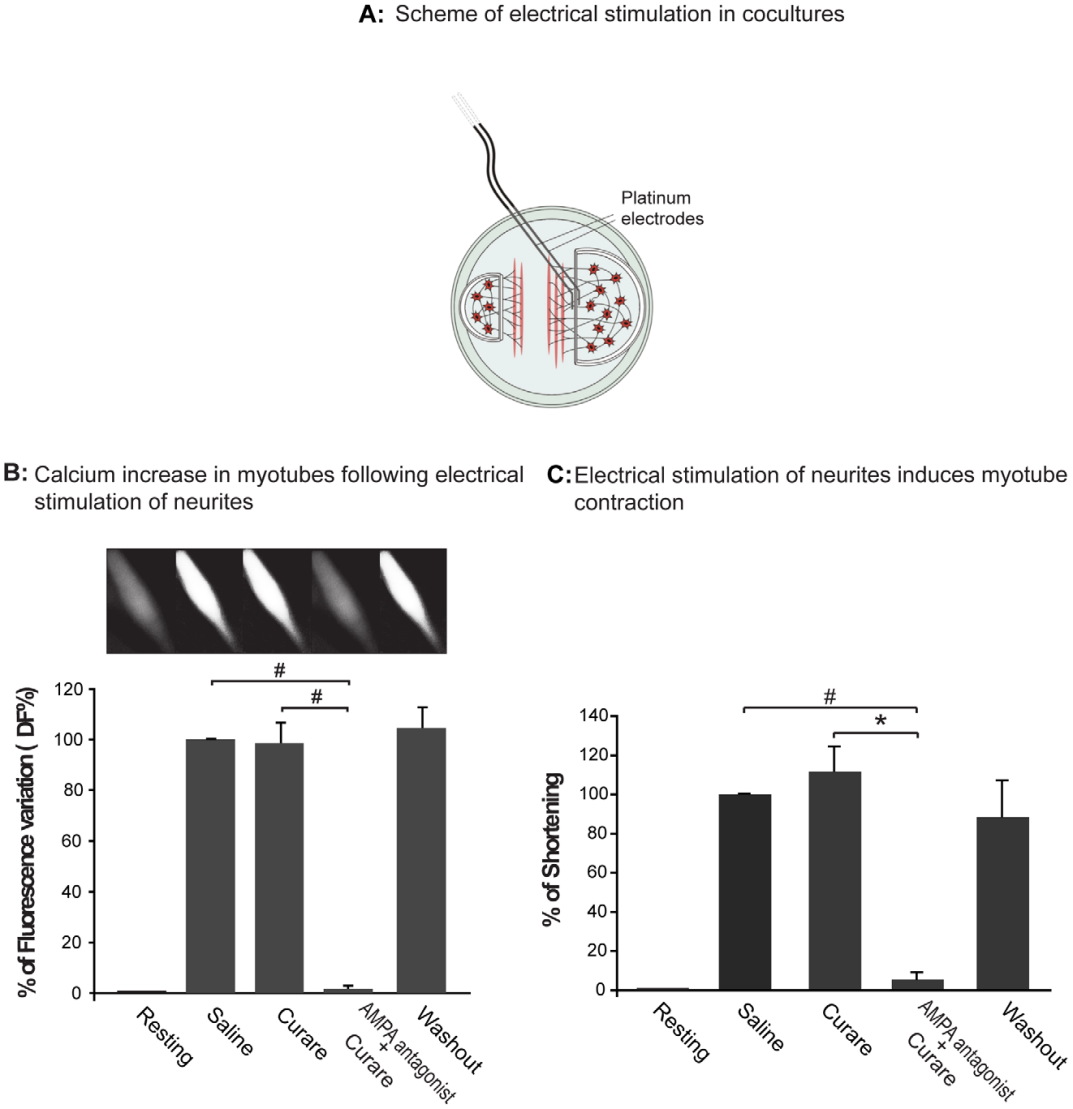
Cortical neurons form fully functional glutamatergic synapses with myotubes. In A a scheme of the coculture plate shows how the stimulus was applied to axons crossing the teflon barrier. Calcium-dependent fluorescence variations (B) and myotube shortening during contraction (C) have been measured during electrical stimulation while myotubes were sequentially bathed in saline, treated with Curare, treated with AMPAR antagonist, and after washout. In B an example of myotube fluorescence is also shown for each condition. In all the experiments the treatment with the AMPAR antagonist GYKI 52466 gave a complete block of calcium transients and myotube shortening, demonstrating that synapses are functional and glutamatergic. N = 3, *p<0,05, #p<0,01 by t-test (paired sample, 2 tailed).

To evaluate whether different glutamatergic neurons are able to form synapses with myotubes, a non-physiological synaptic partner, we cocultured myotubes for 7–9 days with neurons obtained by different brain areas: cortex, cerebellum and hippocampus. In particular, to facilitate the immunofluorescence and electrophysiology studies, we separated the two populations of cells using two half teflon rings soaked in silicon grease and laid down on the bottom of a Petri dish to obtain a Campenot-like chamber, in which neurons were seeded inside the rings, whereas myotubes were grown outside (see the scheme in Figure 1A). In such device, neurons made synaptic contacts with each other but a pool of axons were able to grow across the silicone grease/teflon barrier and reach muscle cells. Preliminary experiments were performed to find the optimal conditions to maintain soma of neurons confined into the ring, allowing only axons to cross the barrier. We obtained this, when the electric resistance between inside and outside the teflon ring was about 15–20 kΩ [28].

**Figure 2:**
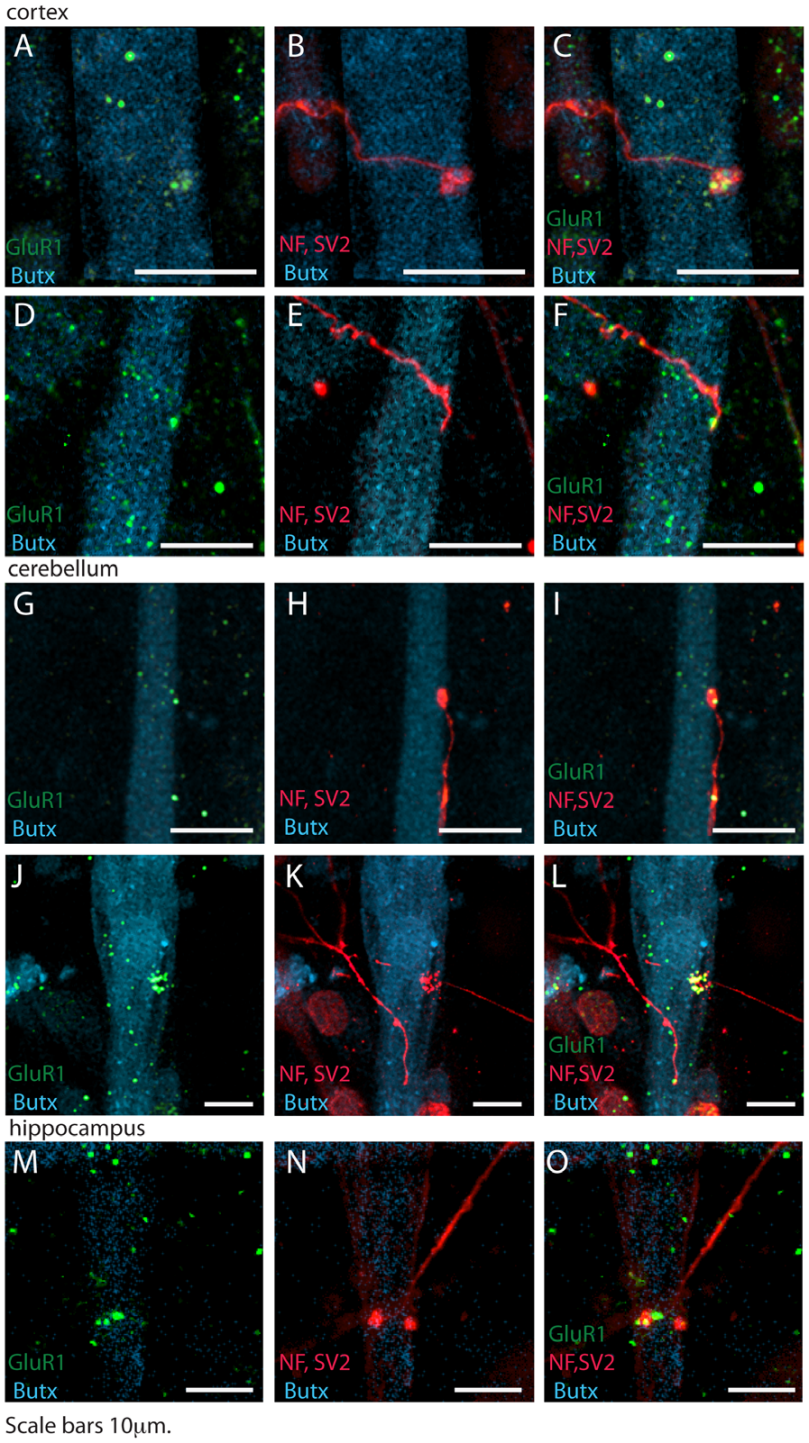
Glutamatergic neurons from different brain areas form synaptic contacts with myotubes in vitro. Confocal images showing synapses between myotubes and glutamatergic neurons from cortex (A–F), cerebellum (G–L) or hippocampus (M–O) after 9 days of coculture and immunostained. AMPARs (GluR1 subunit) are in green, axonal neurofilaments and terminations are in red (NF, SV2), whereas AChRs are in cyan (α-bungarotoxin). AChRs are diffusely distributed on myotube surface, whereas AMPARs form clusters that are often near to the axonal termination. Scale bars 10 µm.

Cocultured cells were fixed and immunostained for AMPA receptors (GluR1 subunit), acetylcholine receptors (AChRs), axons and presynaptic terminations (neurofilaments and synaptophysin respectively). Axons of glutamatergic neurons were able to grow and form contacts with muscle cells even if they were not the physiological postsynaptic partners of glutamatergic neurons (Figure 2 and Figure S1). AMPA receptor clusters were observed at synaptic contacts between muscle and neurons, while AChRs, the physiological postsynaptic receptor in muscle cells, displayed a diffuse distribution. When cocultures of muscle and neurons were stained with antibodies against NMDA receptors (anti-NMDA2B subunit) or GABA receptors (anti-GAD 67), no staining was observed (data not shown).

Moreover, cultures of neurons were immunostained to study evaluate neurotransmitter phenotype in the cell culture. Glutamatergic (vescicular glutamate transporter type-2, anti-VGlut-2), GABAergic (anti glutamic acid decarbossilase, anti-GAD67) and cholinergic (anti choline acetyl transferase, anti-Chat) markers were used. We found that about 95% of the neurons had glutamatergic phenotype.

### Time–course of the expression of glutamate receptors in cocultured myotubes

**Figure 3:**
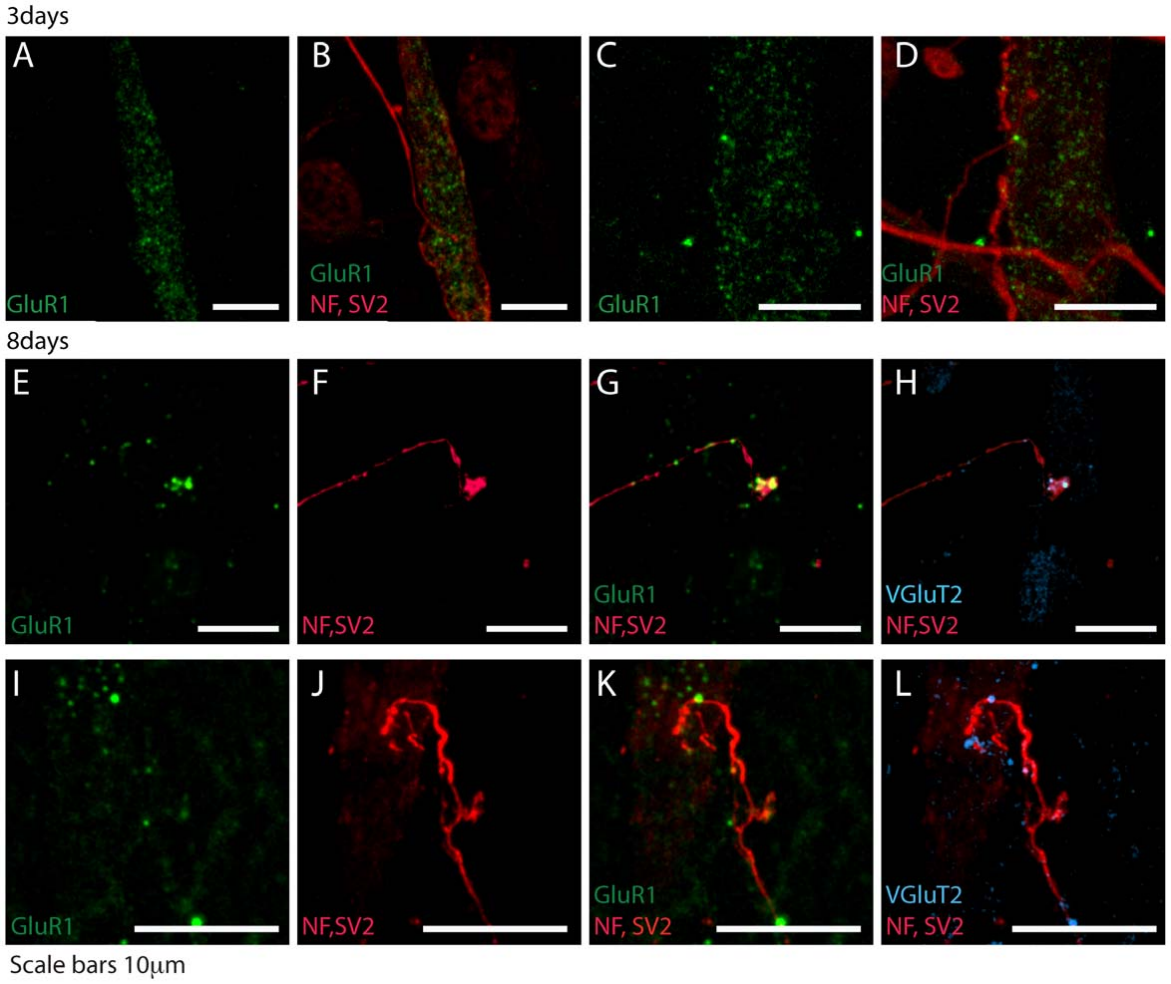
Time course of synaptic contacts formation in cocultures. Examples of cortical neurons cocultured with myotubes for 3 (A–D) and 8 days (E–L), fixed, immunostained and studied by confocal microscopy. AMPARs (GluR1 subunit) are in green, axonal neurofilaments and terminations are in red (NF, SV2). At 3 days AMPARs are diffusely distributed (A, C) and myotubes often receive multiple synaptic contacts (B, D). At 8 days AMPARs form clusters (E, I) near or under the terminations (G, K). The Vesicular Glutamate transporter 2 (VGluT2, blue) confirm that the synaptic contact is glutamatergic (H, L). Scale bars 10 µm.

Next, we examined the time course of GluR1 expression and clustering in cocultured myotubes (Figure 3). To do this, cocultured myotubes were stained for AMPARs, AChRs, and for axons and terminations at 3 and 8 days after myotubes differentiation. At 3 days, AMPA receptors were diffusely expressed by the myotubes, some of which received multiple contacts from axons (Figure 3 A–D). However, at 8 days, most GluR1 were clustered at synaptic contacts, whereas non synaptic AMPA receptors were eliminated (Figure 3 E–L), a behavior resembling the physiological development of AChRs plaques in muscles. In contrast to AMPA receptor clustering, AChRs were found to be distributed on the entire cell surface at both day 3 and day 8 as previously described in cocultured myotubes with cerebellar granule cells [29]. To confirm that contact sites between axons and muscle cells were glutamatergic excitatory synapses, nerve terminals were stained at 8 days with VGluT2 (Figure 3 H–L). This result indicates that most of synaptic sites were glutamatergic synapses.

To further investigate the anatomical distribution of AMPA receptor clusters, we analyzed AMPAR staining on cocultured muscle cells with either hippocampal (20 myotubes observed in 2 plates), cortical (27 myotubes observed in 3 plates), or cerebellar neurons (37 myotubes). We found three types of AMPA receptor profiles as follows: 1) widespread, 2) small clusters diffusely present in the whole cell and 3) bigger clusters co-localized with the synaptic contact. A possible explanation for the co-existence of different AMPARs organization is that in our cocultures neurites take a certain time to grow through the plate and thus they contact myotubes with a different timing, depending on how far from the teflon wall is the muscle cell.

Quantification of AMPARs organization shows that at 9 days the 20% of myotubes did not express AMPARs, the 28% expressed the receptors diffusely, the 50% of the cells displayed clusters. Of these clusters, 20% were localized at synaptic sites.

**Figure 4:**
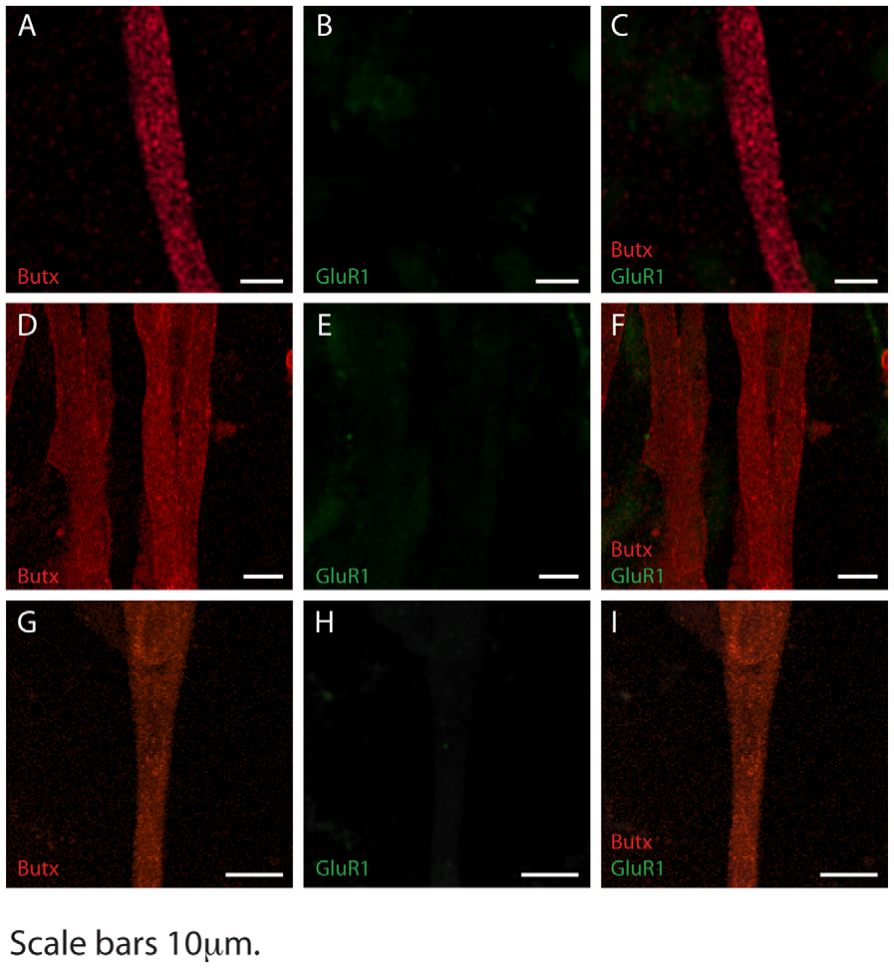
Primary myotubes cultured without neurons do not express AMPARs. Primary myotubes were cultured in absence of neuron and after differentiation, they were treated with the neuronal medium for 1 (A–C), 3 (D–F) and 5 (G–I) days. After fixation and staining for AMPARs (GluR1 subunit, central panels) and AChRs (α-bungarotoxin, left side panels), plates were acquired by confocal microscopy. Right side panels are the overlay of left side and central panels. AchRs are diffusely and widely distributed on muscle surface (A, D, G), whereas no GluR1 positivity was found in all the days examined (B, E, H). Scale bars 10 µm.

Finally, we examined whether myotubes cultured without neurons could express AMPA receptors. We stained primary myotubes without neurons at 1–3-and 5 days post-differentiation (Figure 4) for AMPARs and AChRs. We found that only AChRs were expressed by the myotubes, and these AChRs displayed a diffuse distribution, as usually happens in culture [2].

**Figure 5:**
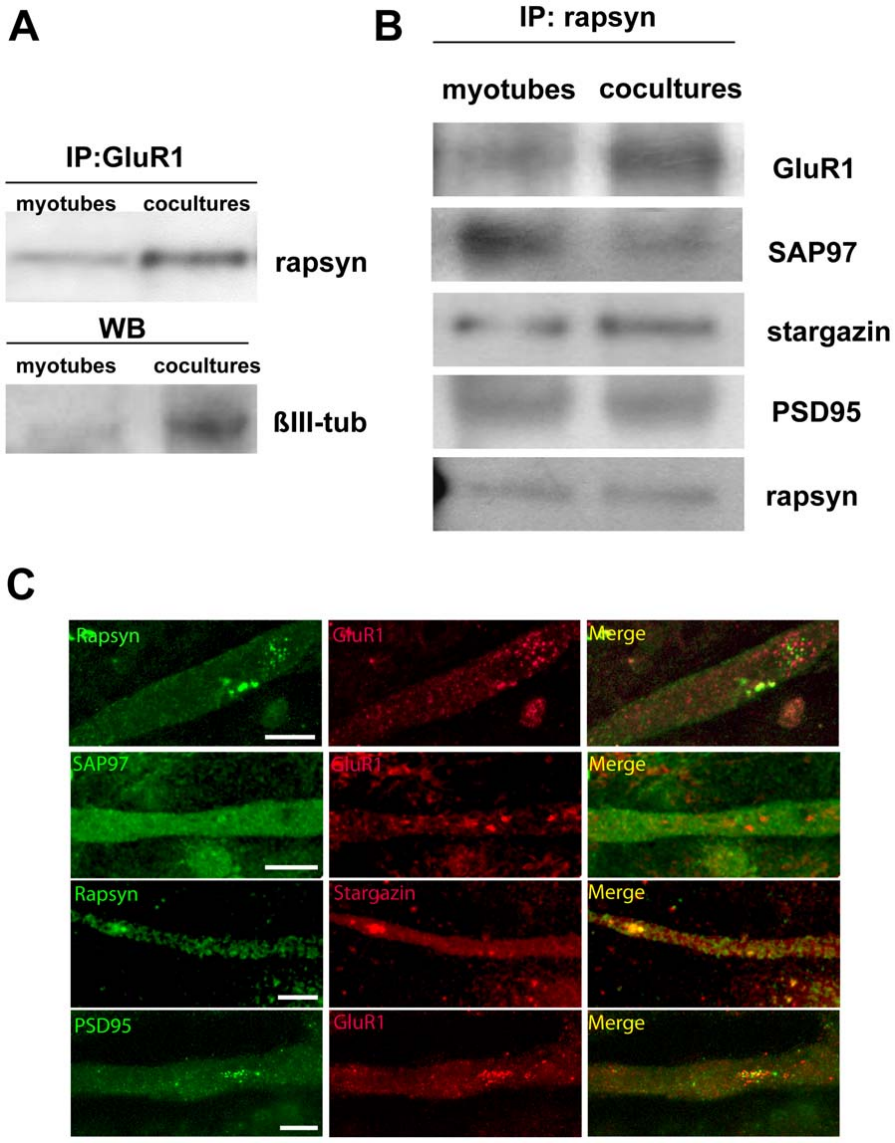
Glutamatergic synaptic components in cell membranes of cocultured myotubes. A: Membrane proteins co-immunoprecipitated by anti-GluR1 antibody were analyzed by immunoblotting using rapsyn antibody. Results show that GluR1 strongly interacted with rapsyn only in cocultured myotubes. Western blot analysis of beta III-tubulin confirmed the presence of neuronal cells only in coculture cell extracts. B: Membrane proteins co-immunoprecipitated by anti-rapsyn antibody were analyzed by immunoblotting using antibodies against GluR1, SAP97, stargazin and PSD95. Results show that both cocoltured myotubes and pure myotubes expressed PSD95, stargazin and SAP97 at the cell membrane. Conversely GluR1 was strongly expressed only in cocultured myotubes. Immunoreactivity of rapsyn-interacting stargazin increased in cocoltured myotubes while that of SAP97 decreased. No change was observed in PSD95 immunoreactivity. C: Confocal images showing the distribution of GluR1, rapsyn, SAP97, stargazin and PSD95 in myotubes cocultured with neurons for 7 days and immunostained. Rapsyn, stargazin and PSD95 colocalize with the receptors, while SAP97 shows a diffuse distribution.

To determine whether AMPA receptors form complexes with scaffold proteins including rapsyn, stargazin, SAP97 and PSD95, we coimmunoprecipitated proteins interacting with rapsyn in membrane preparations from myotubes in cocultures. Rapsyn is the muscle protein anchoring AChRs at the plasmatic membrane and it is essential for clustering AChRs at the postsynaptic apparatus [30], [31]. Membrane extracts from cocultured myotubes with and without neurons were incubated with anti-rapsyn antibody and the immunoprecipitated proteins were analyzed by immunoblotting using antibodies against: 1) GluR1 AMPA receptor subunit, 2) stargazin, the AMPA receptor interacting protein at brain postsynaptic densities, 3) SAP97, the membrane-associated guanylate kinases regulating the AMPA receptor trafficking and 4) PSD95. Immunoblots analyses revealed that GluR1 subunit is immuonoprecipated with rapsyn only in myotubes that are in contact with neurons. However stargazin, SAP97 and PSD95 are immunoprecipated with rapsyn in myotubes cocultured with or without neurons (Figure 5). Concomitantly with the appearance of GluR1, the amount of stargazin also increased in innervated muscle cells while SAP97 decreased, as described previoulsy [25]. No changes were detected in PSD95 levels in myotubes with or without neurons. The post-synaptic localization of AMPA receptors in myotubes cocultured with neurons was further evidenced by the rapsyn immunoreactivity detected in GluR1 immunoprecipitates from membrane proteins (Figure 5). The presence of neuronal terminals in cocultured cells was confirmed by the βIII-tubulin immunoreactivity detected in cell extracts from myotube-neuron template but not in myotube without neurons.

Moreover, to investigate the relative distribution of GluR1, rapsyn, stargazin, SAP97 and PSD95 we immunostained cocultured myotubes. Representative images are shown in Figure 5C. In line with co-immunoprecipitation analysis, we found a colocalization of rapsyn, with both GluR1 and stargazin. The staining of PSD95 colocalized with GluR1 while SAP97 signal showed a diffuse distribution.

### Functional characterization of the synaptic contact between myotubes and cortical glutamatergic neurons

To examine whether glutamatergic synapses in cocultures are functional, we performed calcium imaging experiments and shortening contraction imaging analysis. Cocultured myotubes at 8–9 days, were incubated with Fluo4-AM, a calcium fluorescence probe, and changes in intracellular calcium were monitored during electrical stimulation of axons crossing the teflon walls, as shown in Figure 1A. In each coculture, about 8–10 myotubes were activated by electrical stimulation. Stimulation produced fluorescence transients in basal conditions (i. e. in saline solution) and after AChRs block by curare treatment, meaning that AChRs are not the mediators of synaptic transmission. Importantly, in presence of curare and an AMPAR antagonist (GYKI 52466) fluorescence transients were abolished, whereas after their washing out, calcium transients were restored (see Figure 1B and Movie S1). In control experiments we tested whether the administration of the AMPAR antagonist vehicle affected fluorescence signals (n = 4 cells) and whether the electrical pulse, used to stimulate axons, induced direct activation of myotubes (n = 3 cells). Both experiments didn't produce fluorescence changes in myotubes.

In a different group of cocultures (2 myotubes analyzed) only the AMPAR antagonist was administered and immediately fluorescence transients were blocked. The wash out of GYKI 52466 restored fluorescence increases (Figure S2).

In a motoneuron-myotube synapse, after the stimulation of the axon the calcium increase is followed by sarcomer contraction and subsequent reduction of cell length. The same happened in glutamatergic neuron-myotube cocultures. We quantified the shortening of myotubes on video recordings, by measuring the length variation of cells during electrical stimulation of axons (n = 3 myotubes). The results are plotted in Figure 1C. Axons were stimulated with an electrical pulse of 4–8 V every 2 seconds. In each coculture, 4–6 myotubes, at least, showed contraction. Experimental protocol was the same of calcium imaging experiment. Myotube contraction was observed in saline solution and after curare administration, but not after AMPAR antagonist treatment. The wash out of AMPAR antagonist restored myotube activity (Figure 1C and Movie S2).

In a different group of cocultures we verified that vehicle solution did not affect myotube activity (2 myotubes were studied). To confirm the specificity of AMPA antagonism in inhibiting myotube activity, in another group (2 myotubes analyzed), only GYKI 52466 was administrated. Also in this case the contraction was efficiently blocked.

All these data demonstrate that glutamatergic neurons are able to form a fully functional synapse even with a non physiological postsynaptic partner like myotubes.

## Discussion

The present work shows that glutamatergic neurons have a striking effect on the expression of neurotransmitter receptors of mammalian muscle cells. Particularly, when myotubes were cocultured with glutamatergic neurons, AMPARs were expressed and clustered at synaptic sites, whereas some AChRs remained diffusely distributed on the entire cell surface of the muscle cell and others were aggregated spontaneously into hot spots. Conversely, myotubes cultured without neurons did not express AMPARs but only AChRs. In cocultures we saw that axons contacted myotubes after the third day. At this day AMPARs were widely distributed on the entire surface of muscle cells. Later, at 8th–9th day, AMPARs clustered and colocalized with axon terminals. Our biochemical and immunohistological results show that AMPARs and their associated scaffold proteins present in the brain postsynaptic densities were expressed by muscle cells and form complexes with rapsyn, a component of the scaffolding protein, that it is required to receptor clustering. These data are consistent with previously published work in which skeletal muscles are surgically innervated by spinal glutamatergic fibers [25]. Analysis of GluR1 immunoprecipitates revealed the presence of rapsyn in membrane of myotube-neuron cocultures. In rapsyn-immunoprecipitates we detected GluR1 subunit together with stargazin, a scaffolding protein necessary for the AMPA receptor targeting to the synaptic membrane [32]. Confocal analysis of immunofluorescence staining confirmed the co-localization of rapsyn with either GluR1 or stargazin. Differently from stargazin, SAP97, the key regulator of AMPA receptor trafficking, decreased in cocultured myotubes when compared to pure myotubes cultures, suggesting that in innervated muscle cells GluR1 subunits were stably inserted at the postsynaptic membrane [33]. No change was present in the PSD95 content of the myotubes cultured with and without neurons, also confirming previous evidence [25], [27]. These data are further supported by electrophysiological analysis. In calcium imaging experiments, electrical stimulation of axons induced calcium increases in myotubes that were insensitive to the AChR blocker, curare, but totally prevented by GYKI 52466, the selective blocker of glutamate AMPA receptors [34], [35]. Taken together, these findings expand on previous evidence showing that glutamatergic presynaptic terminals are able to induce a functional postsynaptic membrane structure in muscle cells [25], [27].

Synapse formation is the result of a complex and highly regulated process of membrane and molecular interactions between pre and postsynaptic components. Several works showed the fundamental role of electrical activity, genetic and transcription factors and signaling proteins in the synaptic assembling (see for reviews [2], [20], [36], [37], [38], [39]). However, one of the most intriguing question about synaptogenesis is how the matching between neurotransmitter phenotype and the appropriate postsynaptic receptor is obtained. It has been hypothesized that this process could arise in a number of different ways: 1) the presynaptic terminal induces the expression of the appropriate receptors in postsynaptic membrane independently on the receptors already present on the membrane; 2) the postsynaptic structure activates the expression of a wide range of receptors and the presynaptic element chooses the appropriate receptor.

In support of the former idea, Brunelli et al. have shown that in a particular reinnervation model, in which descending glutamatergic fibers of adult rat were diverted in the spinal cord to skeletal muscle by means of a peripheral nerve graft, the cholinergic synapses switch to the glutamatergic type [25], [26], [27]. Additionally, Spitzer et al. have shown that embryonic muscle cells of Xenopus initially express several classes of transmitter receptors in addition to those for ACh. During normal differentiation and innervation of muscle, the other classes of receptors disappear. Changing the expression of transmitters by altering calcium spike activity leads to retention of the classes of cognate, non cholinergic receptors. Under these conditions, they record glutamatergic, GABAergic, and glycinergic synaptic currents from the skeletal muscle, as well as those mediated by nicotinic AChRs [23], [24]. The results of Brunelli et al. suggest the presence of active mechanisms of induction of receptors, while those of Spitzer et al. indicate a mechanism of selection from a pool of receptors expressed during the development from the target cells. Consistent with these observations, our present studies strongly support the in vivo observations that presynaptic terminal induces postsynaptic structure to express the appropriate neurotransmitter receptor also in target cells that usually don't express its. Surprisingly, in muscles cells glutamatergic neurons induce only AMPARs and not others, suggesting that only some types of receptors are possibly induced in target cells.

Thus, our and Spitzer observations suggest that mammals and amphibians use different mechanisms to regulate the synaptic specificity. An active role of the presynaptic terminal in the mammals was also described in interneuronal synapses in the cerebellum [40] and in the thalamus [41]. For reason unknown to us, previous studies in chick muscle-neuron cocultures failed to show a functional synapses between myotubes and supraspinal glutamatergic neurons. For example, Obata observed end-plate potentials (EPPs) in cocultures of chick myotubes with embryonic chick spinal cord but not with chick cerebellum, cerebrum, superior cervical ganglia or dorsal root ganglia [42].

The mechanisms by which glutamatergic innervations regulate the expression of neurotransmitter receptor and the postsynaptic differentiation are unknown. However, it is possible that the direct contact between neurites and muscle cells may allow physical interactions between membrane surface molecules, which in turn may trigger intracellular signaling cascades, leading to changes in postsynaptic receptor properties. In addition it is possible that neurons may release diffusible factors that promote the clustering of AMPARs. This latter is supported by the observation that AMPARs are found to be distributed on the entire surface of myotubes very distant from growth cone of axons. A candidate anterograde neuronal factor could be Narp [16], [17]. While it remains unknown which factor (s) is released from nerve terminals, our data excluded a role of glial factors since the percentage of glial cells was very low (<2%). Another potential player in postsynaptic differentiation is the neurotransmitter, but observations from Munc18-1-knockout and choline acetyltransferase-knockout mice suggest that the initial assembly of the synapse may proceed without neurotransmitter release [43], [44]. It would be of interest to define signals and molecular mechanisms responsible for glutamatergic synapse formation and maintenance at mammalian NMJ.

The vertebrate NMJ has been characterized as cholinergic (see for a review [2]). However, several reports have described the expression in skeletal muscle cells of neurotransmitter receptors other than the classical nicotinic receptors. Metabotropic glutamate receptors have been described in the adult frog NMJ [45]. Recently, Mays et al. have described the presence of AMPA and NMDA receptor subunits at the postsynaptic membrane of adult NMJ in the mouse quadriceps [46]. The role of these transmitter receptors is unclear, but the investigators suggested that they could take part in the modulation of synaptic activity. In contrast to these studies, our results showed that cultured muscle cells without neurons did not express AMPA and/or NMDA receptors and we did not observe the localization of AMPA and NMDA receptors in the adult NMJ of rat abdominal skeletal muscle [25], [27]. A plausible explanation for the discrepancy with literature could be that differences related to the expression level of receptor subtypes may exist among diverse animal species and muscle types.

## Methods

### Ethics statement

All mice were treated in accordance with the policy of the Italian Ministry of Health and European Community laws on the use of animals in research. The procedures used were approved by the CIRSAL (Interdepartmental Centre of Experimental Research Service) of University of Verona.

#### Neuron-muscle cocultures

Skeletal muscle cells were obtained from E18-E20 C57 mice embryos. Briefly, limbs were collected in Ca^2+^ and Mg^2+^-free Hanks' balanced salt solution. Muscles were removed and cut into small pieces by microforceps. After treatment with trypsin/DNase solutions for 30 min at 37°C and dissociation through a Pasteur pipette, the cell suspension was filtered through a cell strainer (BD Falcon 70 µm). Cells were seeded (1200 cells/mm^2^) in 35 mm Petri dishes (out of the Teflon rings) previously coated with a gelatin and polylisine solution. Culture medium was Dulbecco's Modified Eagle's Medium (DMEM) supplemented with 10% Horse serum, 5% Fetal Calf serum, Glutamine, Penicillin and Streptomycin. At confluency myoblast fusion was induced by serum reduction. Cells were maintained at 37°C and 5% CO_2_ [47]. After 4–5 days, when myotubes start to contract, neurons were seeded inside the Teflon rings.

*Cortical neuron cultures*: cortical neurons were purified from brain of E18 C57 mice embryos. Briefly, fetal mouse brains were removed under a dissecting microscope. After meninges removal, cortices were collected and incubated with trypsin/DNase solutions for 10 min at 37°C. Cells were mechanically dissociated by trituration through a Pasteur pipette and seeded into the Teflon rings (500 cells/mm^2^) in Neurobasal Medium (Gibco), supplemented with B-27 (Gibco), Glutamine, Penicillin and Streptomycin [48].

*Hippocampal neuron cultures*: Hippocampi were collected from brain embryos and processed as described for cortices with only slight differences: the growth medium was added with 0,01 mM glutamate and the chemical dissociation was made without DNAse [48].

*Cerebellar granule cultures*. Cerebella were dissected from P4–6 mice. After meninges removal cerebella were collected in a solution of 35 mM glucose, 2,5 mM HEPES pH 7,4, 4 mM NaHCO_3_ in HBSS (HHGN). Cerebella were washed 3 times with HHGN and incubated in a solution of 10 mg/ml Trypsin, 0,1 mg/ml DNAse in HHGN for 10 min at 37°C. After 3 HHGN washes, the tissue was mechanically dissociated in Basal Medium Eagle (BME) containing 0,1 mg/ml DNAse. The suspension was centrifuged at 200 g for 5 min and the pellet was resuspended in the growth medium (BME supplemented with 10% Bovine Growth Serum, 25 mM KCl, 2 mM glutamine, 100 U/ml penicillin/streptomycin) and triturated again. Cells were plated at a density of 2,5×10^5^ cells/cm^2^ and 10 µM AraC was added to the medium. After 3 days the medium was added with 25 mM glucose [49], [50]. All cocultures were maintained at 37°C in 5% CO_2_ in neuronal medium. Half medium was replaced every 3 days.

### Immunofluorescence analysis

For immunofluorescence studies, cocultures were fixed with 4% paraformaldeyde for 30 min at 4°C. After PBS washes cultures were treated overnight at 4°C with primary antibodies (Abs) and Alexa Fluor conjugated bungarotoxin in blocking solution (2% serum, 2% Bovine Serum Albumin, 0.1% Triton ×100 in PBS). After washes cells were treated with Alexa Fluor conjugated secondary Abs diluted in blocking solution. After washes cells were mounted with glycerol based antifading medium and coverslipped. Images were acquired with Leica SP5 confocal microscope (Leica Microsystem, Manheim, Germany).

Primary Abs used were: anti-GluR1 (Rabbit, 1∶100, Chemicon), anti-VGlut1 and 2 (Rabbit, 1∶1000, SYSY), anti-Neurofilaments SMI312 (mouse IgG1, 1∶1000, Sternberger Monoclonals Incorporated), anti-synaptophysin (IgG1, 1∶500, Chemicon) anti-SAP97 (1∶2000, rabbit polyclonal, Affinity BioReagent Inc. Golden CO), anti-PSD95 (1∶2000, mouse monoclonal, Affinity BioReagent Inc. Golden CO), anti-stargazin (1∶1000, rabbit polyclonal, Upstate) and anti-rapsyn (1∶2000 mouse monoclonal, Affinity BioReagent Inc. Golden CO).

Alpha-Bungarotoxin and the secondary Abs goat anti-rabbit and goat anti-mouse IgG1, were all Alexa Fluor conjugated (1∶1000, Invitrogen) linked to A488, A568 and A647 fluorophores.

### Calcium Imaging

Axons crossing the Teflon wall were stimulated by platinum electrodes and calcium intracellular changes were monitored in myotubes.

Eight-nine day-old cortical/myotube cocultures were incubated for 60 min at 37°C in the recording solution (128 mM NaCl, 4 mM KCl, 1 mM CaCl_2_, 1 mM MgCl_2_, 45 mM sucrose, 10 mM glucose and 0,01 M HEPES; pH 7,4) in presence of Fluo4-AM (Invitrogen) and pluronic F-127 (Invitrogen). After, extracellular Fluo4-AM was removed and cocultures were incubated for 30 min at 37°C with the recording solution to allow a complete de-esterification of intracellular Fluo4-AM. Fluorescence variations were acquired with 20× objective using an inverted microscope (Zeiss Axiovert 35 M) equipped with a cooled CCD camera (Qimaging Retiga-SRV fast, QED InVivo software) at a frequency of 4 frame/s. Excitation of Fluo4 is 488 nm and emission was collected at 510 nm [51].

### Shortening contraction imaging analysis

Axons crossing teflon wall were stimulated using an electrical pulse of 4–8 V of amplitude and 200 µsec of duration every 2 seconds. Coculture medium was replaced with recording solution (128 mM NaCl, 4 mM KCl, 1 mM CaCl_2_, 1 mM MgCl_2_, 45 mM sucrose, 10 mM glucose and 0,01 M HEPES; pH 7,4). Myotubes were stimulated 30 seconds, before the addition of the drugs, to record basal contraction. After D-tubocurarine (AChR antagonist, 2×10^−6^ g/ml) was administrated and finally GYKI 52466 (AMPA receptor antagonist, 10 µM) was added. Contraction was monitored 80 seconds in presence of curare and 60 seconds in presence of AMPA receptor antagonist. In control experiments only GYKI 52466 was administrated and then it was removed. Myotube contraction was recorded by a 20× objective mounted on an inverted microscope (Zeiss Axiovert 35 M). Images were captured with cooled CCD camera (Qimaging Retiga-SRV fast-1394) at an acquisition rate of 4 frames/s using QED InVivo software (Mediacybernetics). Myotube length was evaluated by ImageJ software.

### Co-immunoprecipitation

Only for coimmunoprecipitation studies we didn't use Campenot like chamber. Cocultures were realized seeding cortical neurons over myotubes. Myotubes from pure cultures and myotube-neuronal cultures (3×106 cells) were collected and homogenized by sonication (twice for 10 sec each, at 10 kHz) in 500 µl cold buffer-A containing 0.32 M sucrose, 1 mM HEPES, 1 mM MgCl2, 10 mM NaHCO3, and 0.1 mM phenylmethylsulfonyl fluoride (PMSF) (pH 7.4) in the presence of a complete set of protease inhibitors (Complete; Boehringer Mannheim GmbH, Mannheim, Germany) and phosphatase inhibitors. Homogenates were centrifuged at 13,000 g for 15 min at 4°C and the pellets containing the membrane fractions were separated from cell extracts and suspended in 300 µl cold buffer-B containing 50 mM NaCl, 30 mM triethanolamine, 50 mM NaF, 5 mM EGTA, 5 mM EDTA, 10 mM phospho-nitrophenylphosphate, 50 µM phenylarsine-oxide, 1 mM benzamide, 1 mM N-ethylmaleimide, 1 mM Na-tetrathionate, 1% NP40 Igepal, phosphatase inhibitors and protease inhibitors cocktail. Membrane extracts (40 µg) were precleared in 150 µl buffer-B plus 20 µl protein A/G (Santa Cruz Biotechnology, CA) and rotated for 30 min at 4°C. After centrifugation at 1000 g the supernatant was separated and rotated overnight at 4°C in the presence of rabbit anti-GluR1antibody (1,5 µg, Chemicon) or monoclonal anti-rapsyn antibody (2 µg, Affinity BioReagent Inc. Golden CO). Normal rabbit IgG (Chemicon) was used as control negative antiserum. Thereafter, protein A/G (25 µl) was added and the mixture was rotated for 2 h at 4°C. The beads were washed five times with RIPA buffer (10 mM tris-HCl pH 8, 140 mM NaCl, 0.5% (v/v), Nonidet P-40, 1 mM sodium orthovanodate, 1% protease inhibitor cocktail) and centrifuged at 1000 g for 5 min. Beads were added to SDS loading buffer and boiled for 2 min. After centrifugation, supernatants were immunoblotted using antibodies against: GluR1 (1∶100, rabbit polyclonal, Chemicon), SAP97 (1∶2000, rabbit polyclonal, Affinity BioReagent Inc. Golden CO), PSD95 (1∶2000, mouse monoclonal, Affinity BioReagent Inc. Golden CO), stargazin (1∶1000, rabbit polyclonal, Upstate) and rapsyn (1∶2000 mouse monoclonal, Affinity BioReagent Inc. Golden CO). The βΙΙΙ–tubulin immunoreativity (1∶1000, rabbit polyclonal, Covance) is tested in cell extracts from pure myotubes and myotubes-neuronal cocultures.

## Supporting Information

Figure S1 **Examples of bright field images showing myotubes cocultured with cortical neurons.** The synaptic contact is shown in the left panels and enlarged in the right images. Scale bars 20 µm in A, C, 10 µm in B, F and E, 5 µm in D.
(TIF)

Figure S2 **AMPAR antagonist inhibits calcium increase in myotubes.** Specificity of AMPA antagonist to inhibit myotube activity was shown in calcium imaging experiments in which only GYKI 52466, AMPAR antagonist, was administrated. Fluorescence variations were evaluated during electrical stimulation while myotubes were sequentially bathed in saline, treated with AMPAR antagonist, and after washout. Figure also shows myotube fluorescence signal in each condition. In all experiments GYKI 52466 administration induced a complete inhibition of Ca release highlighting the presence of pure glutamatergic synapse.
(TIF)

Movie S1 Movie shows a myotube during Calcium imaging experiment. Calcium fluorescence increasing, after electrical stimulation of axons, was observed in saline solution and after curare administration. AMPAR antagonist treatment induced block of the fluorescence variations, the washout of the antagonist restored the fluorescence response.
(MOV)

Movie S2 Movie shows an example of glutamatergic innervated myotube that following electrical stimulation of axons showed contraction in saline solution and after curare administration. The administration of AMPAR antagonist quickly inhibited myotube contraction, it was reestablished washing out the antagonists receptors.
(MOV)
