# Correction: Glutamatergic Neurons Induce Expression of Functional Glutamatergic Synapses in Primary Myotubes

**DOI:** 10.1371/annotation/f3df15fa-6983-4410-a27a-f4b8238ba18a

**Published:** 2012-03-29

**Authors:** Michele Ettorre, Erika Lorenzetto, Claudia Laperchia, Cristina Baiguera, Caterina Branca, Manuela Benarese, PierFranco Spano, Marina Pizzi, Mario Buffelli

There was an error in the third affiliation. The correct third affiliation is: 3 National Institute of Neuroscience, Brescia and Verona, Italy.

