# Correction: Glutamatergic Neurons Induce Expression of Functional Glutamatergic Synapses in Primary Myotubes

**DOI:** 10.1371/annotation/e8dec728-6aa1-4581-9f5d-31a39d107f88

**Published:** 2012-03-29

**Authors:** Michele Ettorre, Erika Lorenzetto, Claudia Laperchia, Cristina Baiguera, Caterina Branca, Manuela Benarese, PierFranco Spano, Marina Pizzi, Mario Buffelli

There was an error in the legend of Figure 5. The following text should be added to the end of the legend: Scale bars 10 µm. 

