# Supplementary figures and images for: Glutamatergic Neurons Induce Expression of Functional Glutamatergic Synapses in Primary Myotubes
Source: PLoS One. 2012 Feb 9;7(2):e31451. doi: 10.1371/journal.pone.0031451 (PMC3276509; doi:10.1371/journal.pone.0031451)

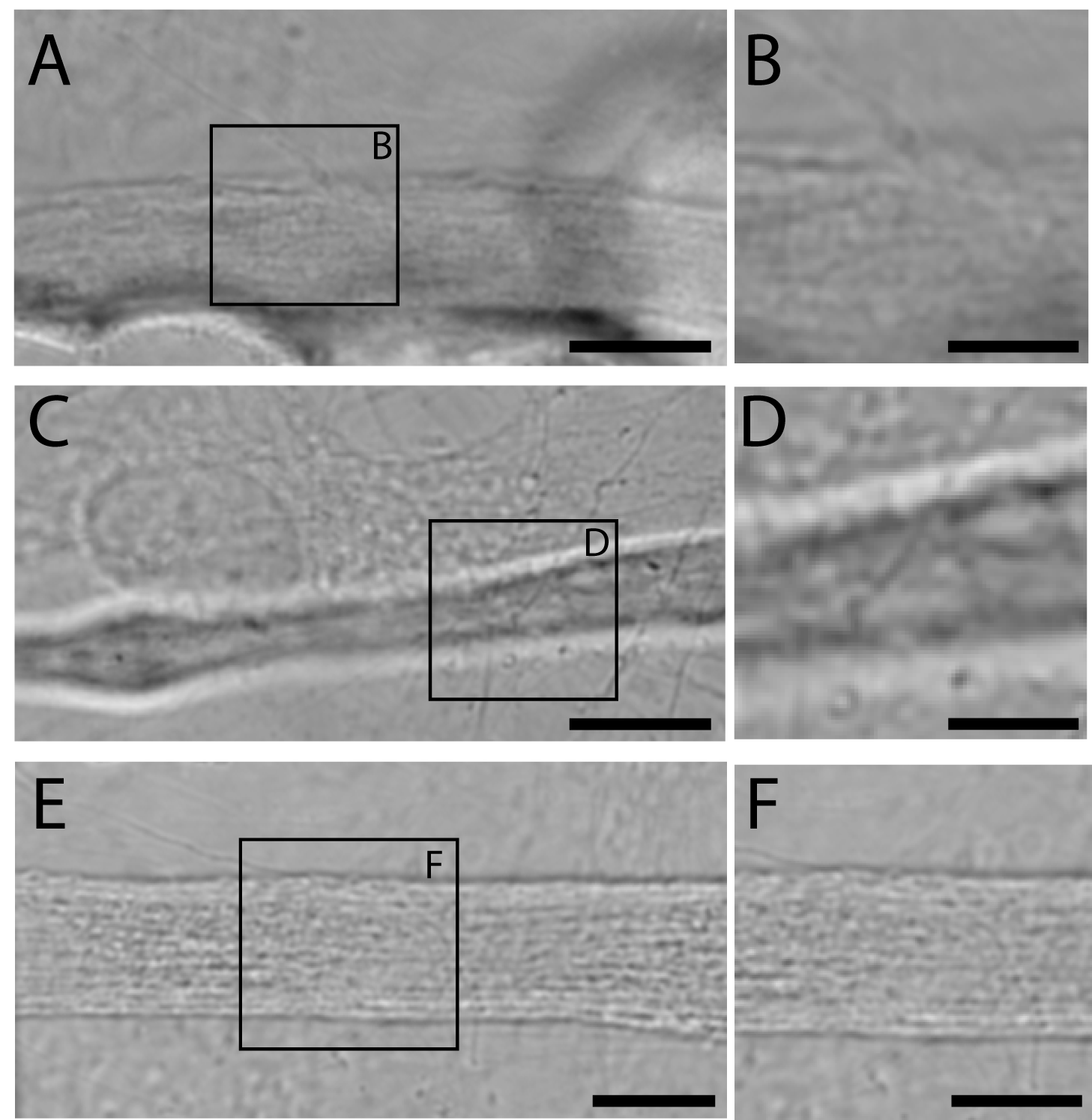

Supplement: Figure S1 — Examples of bright field images showing myotubes cocultured with cortical neurons. The synaptic contact is shown in the left panels and enlarged in the right images. Scale bars 20 µm in A, C, 10 µm in B, F and E, 5 µm in D. (TIF) [file pone.0031451.s001.tif]

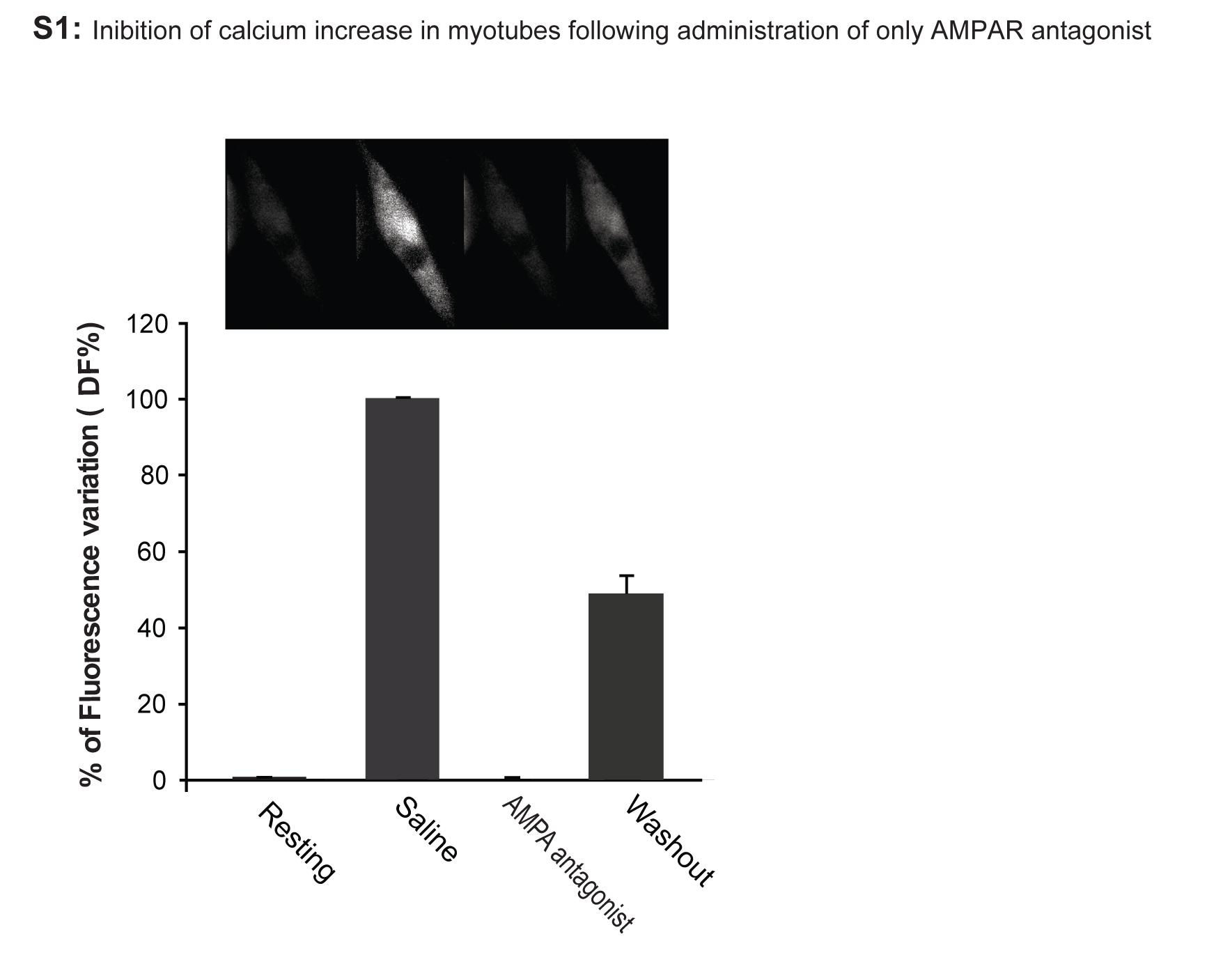

Supplement: Figure S2 — AMPAR antagonist inhibits calcium increase in myotubes. Specificity of AMPA antagonist to inhibit myotube activity was shown in calcium imaging experiments in which only GYKI 52466, AMPAR antagonist, was administrated. Fluorescence variations were evaluated during electrical stimulation while myotubes were sequentially bathed in saline, treated with AMPAR antagonist, and after washout. Figure also shows myotube fluorescence signal in each condition. In all experiments GYKI 52466 administration induced a complete inhibition of Ca release highlighting the presence of pure glutamatergic synapse. (TIF) [file pone.0031451.s002.tif]
